# Diagnostic Accuracy of Dynamic High-Resolution Ultrasonography in Assessing Anterior Disc Displacement in Temporomandibular Joint Disorders: A Prospective Observational Study

**DOI:** 10.3390/healthcare12232355

**Published:** 2024-11-25

**Authors:** Kaili Wang, Chenyang Li, Jinbo Zhou, Jiayin Ren, Meng You

**Affiliations:** State Key Laboratory of Oral Diseases, National Center for Stomatology, National Clinical Research Center for Oral Diseases, Department of Oral Medical Imaging, West China Hospital of Stomatology, Sichuan University, Chengdu 610041, China; wangkaili@scu.edu.cn (K.W.); aaakshei@163.com (C.L.); zhoujinbo@scu.edu.cn (J.Z.); jiayinren@163.com (J.R.)

**Keywords:** temporomandibular joint disorders, anterior disc displacement, dynamic high-resolution ultrasonography, diagnostic efficacy

## Abstract

**Objective:** The objective of this study was to assess the diagnostic efficacy of dynamic high-resolution ultrasonography (HRUS) in detecting anterior disc displacement with reduction (ADDWR) and anterior disc displacement without reduction (ADDWoR) in the temporomandibular joint (TMJ). **Methods:** A total of 144 TMJs was categorized into three groups according to the magnetic resonance imaging (MRI) findings, which served as the reference standard: the normal disc position (NDP) group, the ADDWR group, and the ADDWoR group. Static images of the TMJ in full opening and maximum intercuspal positions, along with dynamic sequences during jaw opening, were obtained utilizing a 14 MHz L-shaped linear array transducer. The diagnostic efficacy of dynamic HRUS for identifying ADDWR and ADDWoR was evaluated in terms of accuracy, sensitivity, specificity, positive predictive value (PPV), negative predictive value (NPV), diagnostic odds ratio (DOR), and the Youden index. **Results:** According to the MRI findings, the NDP, ADDWR, and ADDWoR groups consisted of 42 (29.2%), 47 (32.6%), and 55 (38.2%) TMJs, respectively. HRUS data revealed 54 TMJs (37.5%) in the NDP group, 26 TMJs (18.1%) in the ADDWR group, and 64 TMJs (44.4%) in the ADDWoR group. With MRI as the reference standard, HRUS exhibited a diagnostic accuracy of 71.4%, sensitivity of 51.4%, and specificity of 91.4% for ADDWR. For the ADDWoR, HRUS attained a diagnostic accuracy of 86.5%, sensitivity of 90.0%, and specificity of 82.1%. **Conclusions:** With MRI serving as the reference standard, dynamic HRUS has high diagnostic value for ADDWoR, with better diagnostic accuracy than ADDWR. Ultrasonography has the potential to be used as a highly effective and non-invasive imaging modality for the early screening of ADD in future clinical practice.

## 1. Introduction

Temporomandibular joint disorder (TMD) includes several problems affecting the masticatory muscles, the temporomandibular joint (TMJ), and related structures. The symptoms of TMD depend on the severity of the condition and may include clicking or crepitus noises during jaw movement, limited opening, pain in the TMJ region, masticatory dysfunction, facial asymmetry, and tinnitus—symptoms that can impact an individual’s daily activities, psychosocial functioning, and quality of life [1].

Anterior disc displacement (ADD), the predominant subtype of TMD, is subdivided into anterior disc displacement with reduction (ADDWR) and anterior disc displacement without reduction (ADDWoR). ADDWR refers to the condition where the displaced disc returns to its normal position during jaw movement, usually accompanied by a clicking, popping, or snapping noise [2]. Conservative interventions, such as physical therapy, medication, or occlusal splints, frequently yield positive outcomes and prognoses [3,4]. Conversely, ADDWoR is characterized by the failure of the disc to return to its normal position, leading to persistent joint dysfunction, limited opening, and pain, requiring prompt management [2]. In critical instances, minimally invasive interventions or surgical operations may be necessary, accompanied by a comparatively unfavorable prognosis [3,4,5]. Consequently, the precise diagnosis and distinction between ADDWR and ADDWoR are crucial.

The diagnosis of TMD often entails an amalgamation of history, examination, and imaging procedures, with imaging being crucial for both diagnosis and differential diagnosis [2]. Frequently employed imaging techniques for TMJ problems include conventional radiography, cone-beam computed tomography (CBCT), computed tomography (CT), magnetic resonance imaging (MRI), and ultrasonography (US) [6]. Conventional radiography, CBCT, and CT allow for an effective assessment of hard tissues but provide low resolution for soft tissues. MRI—considered the gold standard for assessing TMJ disc shape and positioning—offers high-resolution images devoid of ionizing radiation [7,8]. Nonetheless, MRI is expensive, time-consuming, and requires a dedicated TMJ surface coil, rendering it impractical for regular application in certain healthcare environments. In contrast, US has emerged as a non-invasive, radiation-free imaging modality that allows for the real-time visualization of joint structures, spatial relationships, and functional interactions between bones, the articular disc, and surrounding muscles. US is especially valuable in cases where rapid diagnostic evaluation is needed or where MRI is impractical due to its cost, availability, or patient contraindications [9,10,11].

High-resolution US (HRUS) has exhibited significant diagnostic precision for ailments such as rheumatoid arthritis and bursitis [10,12]. Previous studies have highlighted the utility of US in assessing TMJ pathologies due to its real-time and non-invasive imaging capabilities, particularly in evaluating soft tissue structures [8,13,14,15,16]. However, its utilization in TMD is still constrained, and relevant studies are scarce. Therefore, this study aimed to establish diagnostic criteria for normal TMJ, ADDWR, and ADDWoR using dynamic HRUS, and to investigate the diagnostic value of dynamic HRUS for ADDWR and ADDWoR.

## 2. Materials and Methods

### 2.1. Data Source

In this study, we recruited patients who attended the West China Hospital of Stomatology, Sichuan University, from August 2022 to August 2023. Informed consent was provided by each participant. Clinical evaluation was conducted in accordance with the Diagnostic Criteria for Temporomandibular Disorders (DC/TMDs) [2]. The inclusion criteria were as follows: (1) age ≥ 18 years; and (2) the existence of at least one of the following symptoms or signs: spontaneous or palpated pain in 1 or both TMJs, masticatory muscle soreness, TMJ noise(s), or limited jaw opening. The exclusion criteria included a history of previous or in-progress TMJ treatment, contraindications to MRI, and patients with any difficulty cooperating with ultrasound examination.

### 2.2. Sample Size Determination

The sample size was calculated based on prior research and statistical considerations. A power analysis was performed using sensitivity and specificity data from previous studies [8,17] on TMD. To detect significant differences in diagnostic performance between HRUS and MRI for ADDWR and ADDWoR, with 80% power and a significance level of 0.05, a minimum of 120 TMJ cases was needed.

### 2.3. Magnetic Resonance Imaging Examination

MRI scans were conducted with 1.5 T or superior equipment equipped with a head-neck coil or TMJ surface coil. Patients were scanned in the supine posture, with their heads oriented first. Images of the left and right TMJs were obtained in the closed-jaw oblique sagittal and oblique coronal orientations, as well as in the full opening oblique sagittal position. ADDWR is diagnosed when, in the maximum intercuspal position, the posterior band of the disc is located anterior to the 11:30 position, the intermediate zone of the disc is anterior to the condylar head, and, on full opening, the intermediate zone of the disc is located between the condylar head and the articular eminence. ADDWoR is identified when the posterior band of the disc remains anterior to the 11:30 position in the full opening and maximum intercuspal positions [2]. The MRI images were evaluated by a TMJ specialist and confirmed by a senior dentomaxillofacial radiologist.

### 2.4. Ultrasonographic Examination

HRUS examinations were performed utilizing a 14 MHz L-shaped linear array transducer (L16-4 HU Ultrasonic Transducer, Resona 5; Mindray, Shenzhen, China) in musculoskeletal (MSK) ultrasonography mode. All examinations were conducted by the same practitioner, who has over 10 years of clinical expertise in US examinations, and the results were confirmed by another experienced ultrasonography specialist. The cumulative image acquisition duration was approximately 10 min for each TMJ.

**Figure 1:**
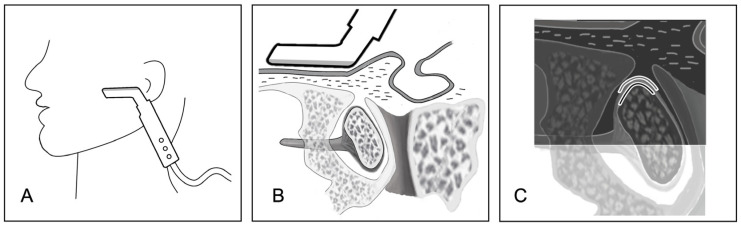
Schematic illustration of the US examination: (**A**) the L-shaped ultrasonographic transducer was positioned transversely anterior to the tragus; (**B**,**C**) the corresponding anatomical schematic and ultrasonographic schematic.

**Figure 2:**
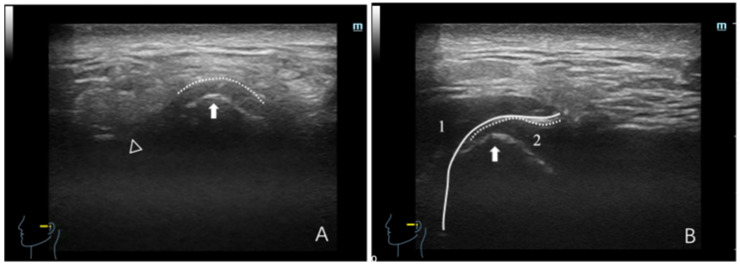
Ultrasonographic image depicting a normal TMJ. (**A**) Maximum intercuspal position; (**B**) full opening position. In panel (**A**), the dotted line denotes the joint capsule, depicted as a hyperechoic (white) line. The white triangle indicates the articular eminence, whereas the white arrow indicates the condyle, with the condyle surface manifesting as a hyperechoic line. In panel (**B**), (1) denotes the masseter, depicted as hypo-to-isoechoic, with white lines outlining the masseter boundary. (2) denotes the articular disc, which appears as a thin hypo-to-isoechoic band, with the dotted line delineating the joint capsule.

Patients were positioned in a supine orientation, and the transducer was positioned transversely above the TMJ region, parallel to the zygomatic arch. The preliminary scan was performed with the mouth closed, maintaining the maximum intercuspal position. The condyle and adjacent soft tissues were assessed from superior to inferior until a distinct sonographic image was obtained (Figure 1). The image was subsequently frozen and documented. Patients were instructed to gradually open their mouth to the furthest extent while the movement of the condyle was monitored in real-time. A static image was captured at the full opening position. Static images (full opening and maximum intercuspal positions) and dynamic films of the jaw-opening process were recorded for each TMJ. During the examination, the transducer maintained contact with the skin without applying pressure to the TMJ and remained in a fixed position. Figure 2 displays a standard US image.

### 2.5. Diagnostic Criteria

This study refines and gives detailed diagnostic criteria for NDP, ADDWR, and ADDWoR based on findings from preliminary trials and the previous literature [15,18,19,20,21].

**Figure 3:**
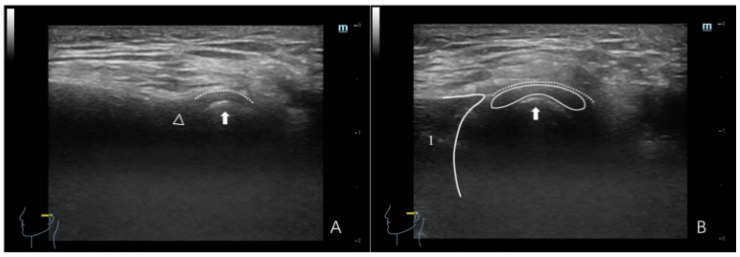
Ultrasonographic image depicting a normal TMJ. (**A**) Maximum intercuspal position; (**B**) full opening position. In panel (**A**), the dotted line denotes the joint capsule, manifesting as a hyperechoic (white) line. The white triangle denotes the articular eminence, whereas the white arrow represents the condyle, where the condyle surface appears as a hyperechoic line. The region between the condyle and the articular eminence corresponds to the articular disc. In panel (**B**), (1) denotes the masseter, depicted as hypo-to-isoechoic, with white lines outlining the masseter boundary. The dotted line denotes the joint capsule, whereas the white arrow indicates the condyle. The articular disc appears as a thin hypo-to-isoechoic band encircling the anterior, superior, and posterior sections of the condyle.

NDP: Essential dynamic HRUS signs comprise seamless, continuous condylar motion without significant changes in the joint space (the distance between the condylar head and the joint capsule). The primary characteristics are as follows: (a) In the maximum intercuspal position, the joint space exhibits a uniform distribution. During movement, the joint space remains consistent, and the condyle demonstrates adequate mobility. On full opening, the condyle tightly aligns with the masseter, and the disc is situated in the posterior-superior region of the condyle (Figure 2). (b) In instances with a diminished condyle, in the maximum intercuspal position, the joint space remains relatively uniform or slightly thickens in the anterior region of the condyle. During movement, there is minimal change in the joint space, and the condyle exhibits adequate mobility. On full opening, the articular disc encircles the anterior, superior, and posterior regions of the condyle (Figure 3).

**Figure 4:**
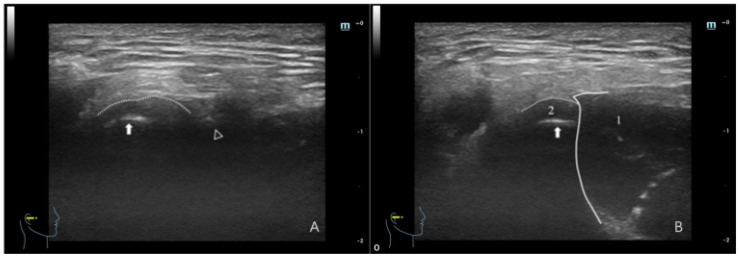
Ultrasonographic image of a TMJ with ADDWR. (**A**) Maximum intercuspal position; (**B**) full opening position. In panel (**A**), the dotted line denotes the joint capsule, depicted as a hyperechoic (white) line. The white triangle denotes the articular eminence, while the white arrow indicates attention to the condyle, with the condyle surface illustrated as a hyperechoic line. The interval between the condyle and the articular eminence pertains to the region of the articular disc. In panel (**B**), (1) denotes the masseter, depicted as hypo-to-isoechoic, with white lines outlining the border of the masseter; and (2) denotes the articular disc, which appears as a thin hypo-to-isoechoic band, with the dotted line illustrating the joint capsule and the white arrow signifying the condyle.

**Figure 5:**
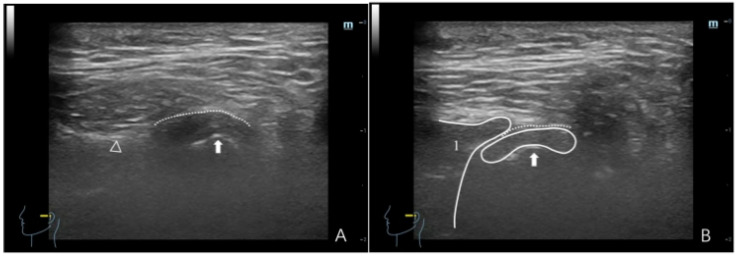
Ultrasonographic image of a TMJ with ADDWR. (**A**) Maximum intercuspal position; (**B**) full opening position. In panel (**A**), the dotted line denotes the joint capsule, depicted as a hyperechoic (white) line. The white triangle indicates the articular eminence, whereas the white arrow indicates the condyle, with the condyle surface manifesting as a hyperechoic line. The interval between them pertains to the articular disc. In panel (**B**), (1) denotes the masseter, depicted as hypo-to-isoechoic, with white lines delineating the masseter border. The dotted line denotes the joint capsule, whereas the white arrow represents the condyle. The articular disc is illustrated as a thin hypo-to-isoechoic band situated anteriorly, superiorly, and posteriorly to the condyle.

**Figure 6:**
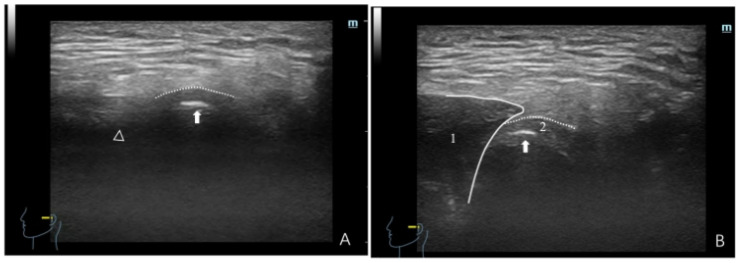
Ultrasonographic image of a TMJ with ADDWR. (**A**) Maximum intercuspal position; (**B**) full opening position. In panel (**A**), the dotted line signifies the joint capsule, illustrated as a hyperechoic (white) line. The white triangle denotes the articular eminence, whereas the white arrow indicates the condyle, where the condyle surface manifests as a hyperechoic line. The interval between the condyle and the articular eminence is occupied by the articular disc. In panel (**B**), (1) denotes the masseter, depicted as hypo-to-isoechoic, with white lines delineating the border of the masseter; and (2) denotes the articular disc, observed as a hypo-to-isoechoic narrow band, with the dotted line indicating the joint capsule and the white arrow directing attention to the condyle.

ADDWR: The principal dynamic HRUS signal is a gradual reduction in the joint space during motion. The primary characteristics are as follows: (a) In the maximum intercuspal position, the anterior joint space is expanded. During movement, the joint space diminishes, indicating that the disc relocates from the anterior position of the condyle to its typical posterior-superior site. Despite the small limitations in condylar motion, it remains operational. On full opening, the condyle is near the masseter, with the disc positioned in the posterior-superior region (Figure 4). In instances of a thinner condyle, the disc encircles the anterior, superior, and posterior parts of the condyle (Figure 5). (b) In the maximum intercuspal position, the joint space is consistently limited. Movement has a distinct transition (narrow-wide-narrow), indicating disc shrinkage. Despite uneven condylar movement, mobility is typically adequate. On full opening, the condyle is tightly aligned with the masseter, and the disc is situated posterior-superior to the condyle (Figure 6).

**Figure 7:**
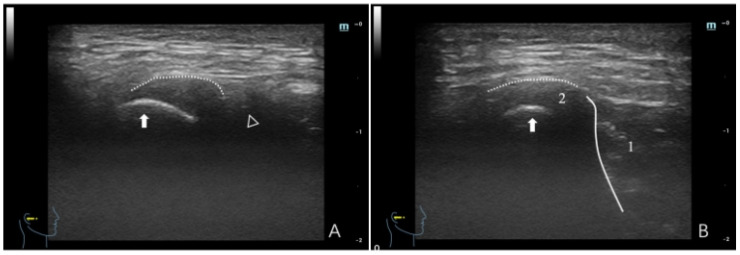
Ultrasonographic image of a TMJ with ADDWoR. (**A**) Maximum intercuspal position; (**B**) full opening position. In panel (**A**), the dotted line denotes the joint capsule, depicted as a hyperechoic (white) line. The white triangle denotes the articular eminence, whereas the white arrow indicates the condyle, where the condyle surface manifests as a hyperechoic line. The interval between the condyle and the articular eminence corresponds to the articular disc. In panel (**B**), (1) denotes the masseter, depicted as hypo-to-isoechoic, with white lines outlining the border of the masseter; and (2) denotes the articular disc, depicted as a thin hypo-to-isoechoic band, with the dotted line indicating the joint capsule and the white arrow directing attention to the condyle.

**Figure 8:**
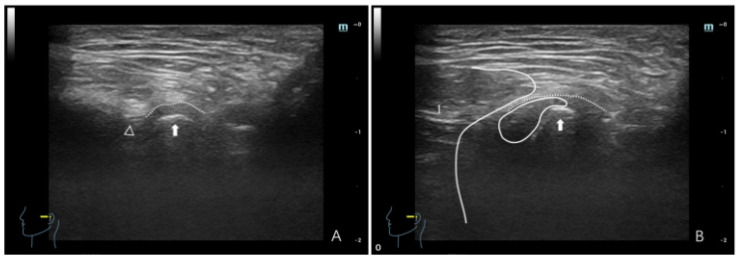
Ultrasonographic image of a TMJ with ADDWoR. (**A**) Maximum intercuspal position; (**B**) full opening position. In panel (**A**), the dotted line denotes the joint capsule, depicted as a hyperechoic (white) line. The white triangle denotes the articular eminence, whereas the white arrow indicates the condyle, where the condyle surface manifests as a hyperechoic line. In panel (**B**), (1) denotes the masseter, depicted as hypo-to-isoechoic, with white lines delineating the border of the masseter. The dotted line denotes the joint capsule, whereas the white arrow indicates the condyle. The white region between the two denotes the articular disc, which is situated anterior to the condyle.

ADDWoR: The principal dynamic HRUS signs of ADDWoR include limited condylar movement, a diminished range of motion, and the continued presence of a hypoechoic or isoechoic region between the condyle and the masseter, even on full opening. The primary characteristics are as follows: (a) In the maximum intercuspal position, the anterior segment of the condyle is significantly thickened, presenting as a hypo-to-isoechoic band. Condylar mobility is markedly impaired during movement, exhibiting minimal or no motion. Alternatively, the condyle may shift marginally, squeezing the hypoechoic region, indicating anterior displacement of the disc. On full opening, the hypoechoic region remains between the condyle and the masseter, indicating that the disc is still displaced anteriorly (Figure 7). (b) In the maximum intercuspal position, the joint space is consistently limited. During motion, the condyle demonstrates partial mobility; nonetheless, on full opening, a hypo-to-isoechoic band persists between the anterior condyle and the masseter (Figure 8). (c) In the maximum intercuspal position, the joint space is consistently limited (Figure 8A). Condylar mobility is significantly constrained during movement, exhibiting minimal to no motion.

### 2.6. Statistical Examination

The MRI data served as the reference standard for evaluating the diagnostic efficacy of dynamic HRUS. Each TMJ was regarded as a statistical entity. Categorical data are expressed as frequencies and percentages, with significance testing conducted via the chi-square test. The diagnostic efficacy of HRUS for detecting ADDWR and ADDWoR was assessed using critical performance metrics, including accuracy, sensitivity, specificity, positive predictive value (PPV), negative predictive value (NPV), positive likelihood ratio (LR+), negative likelihood ratio (LR-), diagnostic odds ratio (DOR), and Youden’s index.

Statistical significance was established at 5% (*p* < 0.05) for all analyses. All analyses were conducted utilizing SPSS 26.0 (IBM Corp., Armonk, NY, USA) or Python 3 (for point-biserial correlation; Python Software Foundation, Wilmington, DE, USA). Both ultrasonography technologists and data analysts were unaware of the MRI results until the completion of data processing.

## 3. Results

### 3.1. Diagnostic Outcomes of HRUS

**Figure 9:**
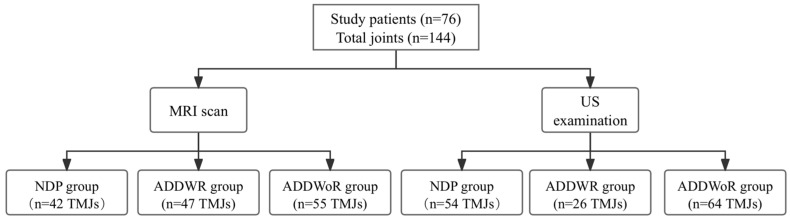
Flowchart of the TMJ inclusion process.

A flow chart illustrating the participant inclusion process is displayed in Figure 9. This study involved 76 patients, encompassing 144 TMJs, treated at the West China Hospital of Stomatology, Sichuan University, from March 2022 to March 2023. Eight more TMJs were removed due to unilateral or bilateral joint pathologies, including disc perforation, disc adhesion, or synovial chondromas. The patients’ ages varied from 18 to 60 years, with a mean age of 28.5 years and a male-to-female ratio of 1:5.3.

The MRI detected 42 TMJs (29.2%) as NDP, 47 TMJs (32.6%) with ADDWR, and 55 TMJs (38.2%) with ADDWoR. According to the previously established diagnostic criteria, US identified 54 TMJs (37.5%) as NDP, 26 TMJs (18.1%) as ADDWR, and 64 TMJs (44.4%) as ADDWoR (Table 1). Chi-square analysis revealed a substantial overall disparity between the MRI and US data (χ^2^ = 8.222, *p* = 0.016). A notable distinction was identified between the NDP group and the ADDWR group (χ^2^ = 7.082, *p* = 0.008), although no significant difference was detected between the NDP and ADDWoR groups (χ^2^ = 0.131, *p* = 0.718).

**Figure 10:**
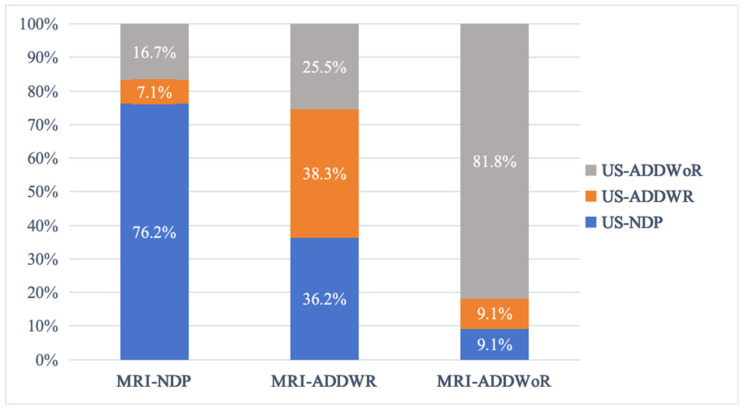
Bar chart depicting the findings of the US examination.

Of the 47 TMJs diagnosed with ADDWR via MRI, 17 were erroneously labeled as NDP via US, 12 as ADDWoR, and 18 were accurately recognized as ADDWR, yielding a concordance rate with MRI of 38.3%. Among the 55 TMJs identified with ADDWoR via MRI, 5 were erroneously classified as NDP via US, 5 as ADDWR, and 45 were correctly identified as ADDWoR, resulting in a concordance rate of 81.8% with MRI (Figure 10).

### 3.2. Diagnostic Efficacy of Dynamic HRUS

The efficacy of dynamic HRUS in identifying ADDWR was evaluated against MRI as the reference standard, utilizing the fourfold table for diagnostic testing (Table 2). The diagnostic accuracy, sensitivity, specificity, positive predictive value (PPV), and negative predictive value (NPV) were 71.4%, 51.4%, 91.4%, 85.7%, and 65.3%, respectively. The positive likelihood ratio (LR) was 5.98 and the negative LR was 0.532, resulting in a diagnostic odds ratio (DOR) of 11.294 and a Youden index of 0.428. The diagnostic accuracy of the ADDWoR was 86.5%, with a sensitivity of 90.0%, a specificity of 82.1%, a positive predictive value of 86.5%, and a negative predictive value of 86.5%. The positive likelihood ratio (LR) and negative likelihood ratio (LR) were 5.027 and 0.123, respectively, with a diagnostic odds ratio (DOR) of 41.143 (Table 3) and a Youden index of 0.721. All diagnostic performance parameters for the ADDWoR, except for specificity, surpassed those for the ADDWR (Table 4).

## 4. Discussion

Recent advancements in imaging technologies have significantly enhanced the diagnostic and therapeutic approaches for TMD. HRUS, a non-invasive modality capable of real-time dynamic examination, has been demonstrated to possess substantial clinical value. The increasing availability of portable ultrasonography devices has facilitated the transition of HRUS from specialized diagnostic centers to more accessible chairside screening and immediate diagnostic applications, thereby improving its flexibility and clinical utility [14,19,22]. While MRI remains the gold standard for soft tissue evaluation, HRUS, due to its portability, real-time imaging, and cost-effectiveness, has become a viable alternative for dynamic TMJ function evaluation [8,23,24].

Moreover, HRUS offers considerable advantages in US-guided interventions and real-time therapeutic examinations. Its ability for real-time imaging enhances both the precision and safety of minimally invasive procedures, such as intra-articular injections, by allowing clinicians to visualize and adjust interventions in real-time [25,26]. Additionally, HRUS facilitates dynamic feedback during treatment, enabling continuous monitoring of joint structures and real-time adaptation of therapeutic strategies. This real-time feedback mechanism not only improves the procedural accuracy, but also mitigates the risk of complications and accelerates patient recovery, particularly in cases involving complex joint pathologies [19,27].

Prior research on the use of US for diagnosing ADD in the TMJ has predominantly depended on static images obtained in full opening and maximum intercuspal positions. Due to the variability in the US scanning protocol, the US imaging performance of TMJ structures is inconsistent. Furthermore, the diagnostic criteria for ADD via US lack standardization, leading to a broad range of diagnostic sensitivity and specificity. In 1997, Emshoff et al. [19] innovated the use of dynamic US for detecting TMJ internal derangements, showing its capacity for the real-time viewing of TMJ components during jaw movement and facilitating repeated functional examinations. Almeida et al. [28] observed that the ultrasonographic resolution employed in the TMJ region varied from 5 to 20 MHz; nevertheless, studies indicate that HRUS surpassing 12 MHz provides enhanced resolution, hence augmenting tissue differentiation and diagnostic precision [29]. Moreover, compared with static low-resolution US, dynamic HRUS enhances both the sensitivity and specificity of TMJ disc displacement diagnosis.

The clinical utilization of MSK US has markedly increased in recent years, mostly owing to its ability to provide real-time imaging of TMJs, discs, muscles, and tendons during functional activities [30]. Researchers have reported that high-resolution linear transducers, when utilized in MSK mode, yield crisper echogenic images of joints [31]. Thus, this investigation utilized dynamic HRUS in MSK mode, employing a scanning protocol established in our prior research [8], in order to assess its diagnostic accuracy for ADDWR and ADDWoR.

A prevalent clinical manifestation of ADDWR is joint clicking noise, resulting from disc repositioning to its natural alignment with the condyle and articular eminence during jaw opening or functional movement [32]. We hypothesized that dynamic US could capture the disc’s real-time “snapping back” mechanism during condylar forward movement, as evidenced by the hypo-to-isoechoic region transitioning from the anterior to the posterior-superior aspect of the condyle in the full opening position or by alterations in the joint space. In ADDWoR, patients generally exhibit limited jaw opening and, according to diagnostic criteria [2], ADDWoR can be categorized as either with or without limited opening. The radiological diagnostic criteria for ADDWoR specify that the anteriorly displaced disc remains positioned anterior to the condyle during jaw opening. Consequently, we posited that, in instances of ADDWoR, no substantial alteration would be evident in the hypo-to-isoechoic region anterior to the condyle during jaw opening.

Although numerous studies [15,19,21,33,34] have utilized dynamic US for the diagnosis of ADD, the majority of diagnostic criteria have depended exclusively on static images captured in full opening and maximum intercuspal positions, neglecting the examination of the positional relationship between the disc and condyle during movement. Conversely, the criteria employed have been vague, with descriptions limited to the observation of whether the disc reverted to its natural position during jaw opening. Despite the favorable outcomes reported in these investigations, the application of US for evaluation of the disc-condylar connection in clinical practice continues to pose difficulties. This work enhances and broadens the diagnostic criteria for dynamic US with respect to NDP, ADDWR, and ADDWoR, considering individual anatomical variances to further improve the diagnostic accuracy.

Most prior research on the diagnostic efficacy of dynamic US for ADD has predominantly concentrated on comparing diagnostic parameters in full opening and maximum intercuspal positions [18,19,21,35], with a limited number of studies examining ADDWR and ADDWoR. Kaya et al. [15] have reported that the diagnostic accuracy, sensitivity, and specificity for ADDWR were 57%, 70%, and 38%, respectively, whereas those for ADDWoR were 76%, 50%, and 89%, respectively. Kader et al. [35] similarly reported superior diagnostic accuracy, sensitivity, and specificity for ADDWoR compared to ADDWR. Additional research [36] indicates that dynamic US typically demonstrates superior diagnostic accuracy for ADDWoR compared to ADDWR, aligning with the results of the current investigation.

This study enhanced the diagnostic criteria and further categorized ADDWoR based on the dynamic HRUS findings, achieving exceptional sensitivity and specificity in the diagnosis of ADDWoR, with a Youden index exceeding that reported in most prior studies [15,17], indicating superior diagnostic efficacy and increased reliability. The sensitivity for ADDWR was markedly inferior to that of ADDWoR, potentially attributable to the variability in disc displacement in ADDWR cases, where the displaced disc may demonstrate deformation or folding, hindering the precise observation of the disc’s “snapping back” phenomenon through dynamic US. This probably resulted in a reduced sensitivity in the diagnosis of ADDWR and an increased likelihood of overlooked diagnosis.

Despite the overall robust performance of dynamic HRUS for ADDWoR, some extreme instances of ADDWoR without limited opening were noted. In some instances, significant disc displacement produced ultrasonographic images of the disc-condyle connection in both the full opening and maximum intercuspal positions, which nearly mirrored those of normal TMJs. Furthermore, the seamless motion of the condyle during jaw opening resembled that of healthy TMJs, resulting in a false-negative diagnosis. In particular, five patients who were misdiagnosed because of this condition were identified in this study. Subsequent research should investigate other scanning methodologies and image processing techniques to address these diagnostic problems more effectively.

It should be noted that this study has several limitations. First, this study did not perform a direct comparison of the diagnostic efficiency between dynamic and static HRUS, which may limit a comprehensive assessment of the unique advantages of dynamic imaging. This comparative analysis is intended for exploration in subsequent research. Additionally, factors such as patient age, gender, and history of TMJ dysfunction, examiner dependency, and equipment variability were not fully accounted for, which may introduce bias. Future research should focus on multicenter clinical trials with larger sample sizes, incorporating a broader range of patient variables to increase the generalizability and reliability of the findings.

## 5. Conclusions

This research utilized a diagnostic experimental design, employing MRI as the reference standard. The reported findings demonstrate that dynamic HRUS has significant diagnostic value for ADDWoR, exhibiting superior diagnostic accuracy relative to that of ADDWR. Dynamic HRUS is an effective and non-invasive imaging modality suitable for expediting the chairside examination of TMJ anterior disc displacement.

## Figures and Tables

**Table 1:** Statistical description of the MRI and US examination results.

	MRI		US		*p* Value
	Frequency	Percentage (%)	Frequency	Percentage (%)	
NDP	42	29.2	54	37.5	
ADDWR	47	32.6	26	18.1	0.008
ADDWoR	55	38.2	64	44.4	0.718
Total	144	100	144	100	0.016

MRI, magnetic resonance imaging; US, ultrasonography; NDP, normal disc position; ADDWR, anterior disc displacement with reduction; ADDWoR, anterior disc displacement without reduction.

**Table 2:** Comparison of US and MRI diagnostic results for ADDWR.

US Results	MRI Results		Total
	ADDWR	NDP	
ADDWR	18	3	21
NDP	17	32	49
Total	35	35	70

MRI, magnetic resonance imaging; US, ultrasonography; NDP, normal disc position; ADDWR, anterior disc displacement with reduction.

**Table 3:** Comparison of US and MRI diagnostic results for ADDWoR.

US Results	MRI Results		Total
	ADDWoR	NDP	
ADDWoR	45	7	52
NDP	5	32	37
Total	50	39	89

MRI, magnetic resonance imaging; US, ultrasonography; NDP, normal disc position; ADDWoR, anterior disc displacement without reduction.

**Table 4:** Diagnostic performance of US for ADDWR and ADDWoR.

	ADDWR (%)	95%CI for ADDWR	ADDWoR (%)	95%CI for ADDWoR
Sensitivity	51.4	34.3–68.3	90.0	77.4–96.3
Specificity	91.4	75.8–97.8	82.1	65.9–91.9
PPV	85.7	62.6–96.2	86.5	73.6–94.0
NPV	65.3	50.3–77.9	86.5	70.4–94.9
Accuracy	71.4	60.8–82.0	86.5	79.4–93.6
DOR	11.294	2.91–43.85	41.143	11.98–141.31

ADDWR, anterior disc displacement with reduction; ADDWoR, anterior disc displacement without reduction; CI, Confidence interval; PPV, positive predictive value; NPV, negative predictive value; DOR, diagnostic odds ratio.

## Data Availability

The data presented in this study are available to all researchers.
